# The role of miR-497-5p in myofibroblast differentiation of LR-MSCs and pulmonary fibrogenesis

**DOI:** 10.1038/srep40958

**Published:** 2017-01-18

**Authors:** Xiang Chen, Chaowen Shi, Cong Wang, Weilin Liu, Yanhong Chu, Zou Xiang, Kebin Hu, Ping Dong, Xiaodong Han

**Affiliations:** 1 Immunology and Reproduction Biology Laboratory & State Key Laboratory of Analytical Chemistry for Life Science, Medical School, Nanjing University, Nanjing, Jiangsu 210093, China; 2 Jiangsu Key Laboratory of Molecular Medicine, Nanjing, 210093, China; 3 Department of Health Technology and Informatics, Faculty of Health and Social Sciences, The Hong Kong Polytechnic University, Hung Hom, Kowloon, Hong Kong, China; 4 Department of Medicine, Division of Nephrology, Penn State University College of Medicine, Hershey, 17033, Pennsylvania, USA

## Abstract

Idiopathic pulmonary fibrosis (IPF) is a chronic, progressive and fatal fibrotic lung disease characterized by profound changes in stem cell differentiation, epithelial cell phenotypes and fibroblast proliferation. In our study, we found that miR-497-5p was significantly upregulated both during myofibroblast differentiation of lung resident mesenchymal stem cells (LR-MSCs) and in the lung tissues of a pulmonary fibrosis model. In addition, as determined by luciferase assays and Western blot analysis, reversion-inducing cysteine-rich protein with kazal motifs (*Reck*) was identified to be one of the target genes of miR-497-5p, and Reck could suppress the expression of matrix metalloproteinase-2 (Mmp2) and Mmp9, which could activate latent transforming growth factor-β1 (TGF-β1). To test the potential therapeutic significance of this miRNA, we modulated the expression of miR-497-5p in LR-MSCs and relevant animal models. The results demonstrated that upregulation of miR-497-5p could induce LR-MSCs to differentiate into myofibroblasts and promote pulmonary fibrogenesis, while inhibition of its expression could effectively retard these processes. In conclusion, our work supports that controlling pulmonary fibrogenesis via inhibition of miR-497-5p expression may provide a potential therapeutic strategy for IPF.

Idiopathic pulmonary fibrosis (IPF), which is featured by deterioration of respiratory functions clinically, is one of the most common types of interstitial lung diseases. The pathologic features of IPF include heterogenic injury and fibrosis, fibroblast/myofibroblast accumulation beneath flattened alveolar epithelial cells, and inflammation^1^,^2^. And its morbidity and mortality have ever been estimated to be 42.7 per 100,000 and 16.3 per 100,000, respectively^3^. To date, pulmonary fibrosis has been demonstrated to be associated with multiple conditions including exposure to environmental agents, connective tissue disease, ionizing radiation, and certain medications. However, the cause of IPF is still unknown.

IPF is mainly characterized by the activation of fibroblasts and formation of myofibroblastic foci, alveolar epithelial cell injury, and excessive accumulation of extracellular matrix (ECM) in the lung parenchyma^4^,^5^,^6^. In the progress of IPF, ECM components are deposited, removed and remodeled, which enables cell migration, neovascularization, and restructuring of the alveolar-capillary^7^. The ECM is one of the most important cellular and tissue function regulators in the body^8^. Matrix metalloproteinases (MMPs), a family of over 24 zinc-dependent endopeptidases, are capable of degrading any component of the ECM^9^. Dysregulation of MMP activity results in functional alterations and tissue damage^10^. In lungs with usual interstitial pneumonia (UIP), MMP1, MMP2 and MMP9 are highly expressed. These members of the MMP family may be involved in the deregulation of the synthesis and degradation of ECM proteins, which can lead to enlarged ECM deposition in fibrosis^11^. In addition, MMP-mediated degradation of ECM is proposed as a physiological mechanism for the release and activation of latent transforming growth factor (TGF)-β by cleaving its latent associated peptide^12^,^13^. TGF-β, as a key profibrotic agent, is expressed by multiple structural and inflammatory cells in the inflamed airway, including epithelial cells, macrophages, mast cells and fibroblasts^14^. Thus, if we could effectively command dysregulated MMPs, it may provide a new strategy for the therapy of IPF.

microRNAs (miRNAs) are small noncoding RNAs that are directly associated with the developmental process of cancer, diabetes, cardiovascular disease, and lung disease through regulating gene expression^15^,^16^,^17^,^18^,^19^. To date, a large number of miRNAs have been reported to play key roles in the development of IPF. Pandit *et al*. first reported that let-7d is mainly localized to the alveolar epithelium in normal lungs, but is significantly decreased in IPF lungs^15^. miR-21, which was first identified as an oncogenic miRNA targeting many tumor suppressor genes^20^, has been found to be highly increased in myofibroblasts, epithelial cells, as well as the cells surrounding the fibrotic foci of human IPF lungs^21^,^22^.

In our study, we observed that miR-497-5p was significantly higher during the myofibroblast differentiation of lung resident mesenchymal stem cells (LR-MSCs). And we also verified that miR-497-5p could target the 3′-UTR of reversion-inducing cysteine-rich protein with kazal motifs (*Reck*) which could suppress Mmp2 and Mmp9 synthesis and secretion, and impact on ECM integrity^23^,^24^. We further investigated whether this regulation was conserved in mice. In a bleomycin (BLM)-induced pulmonary fibrosis model, miR-497-5p was upregulated, and suppressing miR-497-5p expression using a lentiviral agent *in vivo* reduced the expression of fibrotic markers Mmp2, Mmp9 and Tgfb1 via enhancing the expression of Reck, suggesting augmented *in vivo* lung repair. These data reveal a potential new therapeutic approach for IPF and an intimate interplay among miR-497-5p, Reck, MMPs, TGF-β1 and pulmonary fibrosis.

## Results

### miRNAs are differentially expressed in TGF-β1-treated LR-MSCs

**Figure 1:**
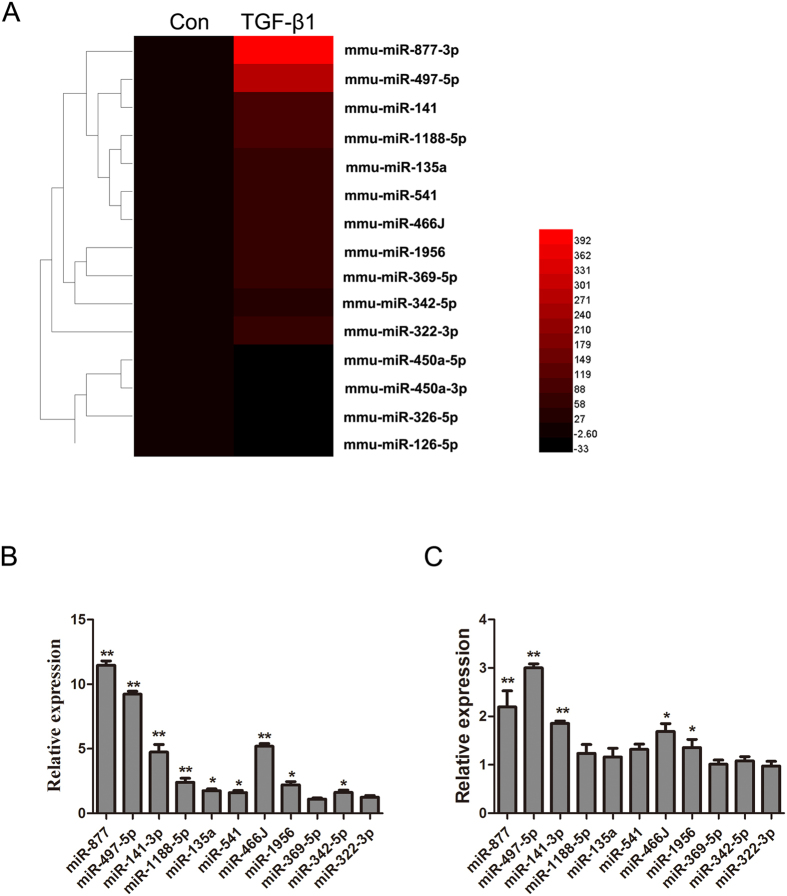
miR-497-5p is upregulated in the myofibroblast differentiation of lung resident mesenchymal stem cells (LR-MSCs) and the lung tissues from a mouse pulmonary fibrosis model. (**A**) A heat map represents the differentially expressed miRNAs in TGF-β1-treated LR-MSCs at day 7. The upregulated miRNAs are indicated in progressively brighter shades of red, and the down-regulated miRNAs are indicated in progressively darker shades of black. (**B**) Quantitative real-rime polymerase chain reaction analysis validation of the microarray results from LR-MSCs administered with TGF-β1 for 7 consecutive days. The data are expressed as the mean ± SD. *P < 0.05, **P < 0.01. (**C**) The mice were injected intratracheally with either saline or bleomycin, and sacrificed on day 14 after injection (n = 6 mice in each group). The 11 miRNAs were also investigated in a bleomycin-induced pulmonary fibrosis model as determined by the quantitative real-rime polymerase chain reaction analysis.

To determine the differentially expressed miRNAs during the myofibroblast differentiation of LR-MSCs, we performed a miRNA microarray with total RNAs isolated from LR-MSCs harvested on day 7 after TGF-β1 treatment. A total of 299 miRNAs were significantly differentially expressed (2^−ΔΔCT^ > 2-fold or < −2-fold)^25^. Among the significantly increased miRNAs, 11 (miR-877-3p, miR-497-5p, miR-141-3p, miR-1188-5p, miR-135a, miR-541, miR-466J, miR-1956, miR-369-5p, miR-342-5p, miR-322-3p) were upregulated over 50 folds, and 4 (miR-450a-5p, miR-450a-3p, miR-326-5p, miR-126-5p) were downregulated over 10 folds (Fig. 1A). To validate the results of miRNA profiling, we confirmed the differential expression of these miRNAs via Q-PCR both in LR-MSCs treated with TGF-β1 and lung tissues from mice that had received BLM intratracheally. We found that miR-497-5p was highly expressed, especially in BLM treated lung tissues (Fig. 1B and C). Thus, miR-497-5p was chosen for further investigation in this study.

### Reck is suppressed in TGF-β1-treated LR-MSCs and the lung tissues of a pulmonary fibrosis model

**Figure 2:**
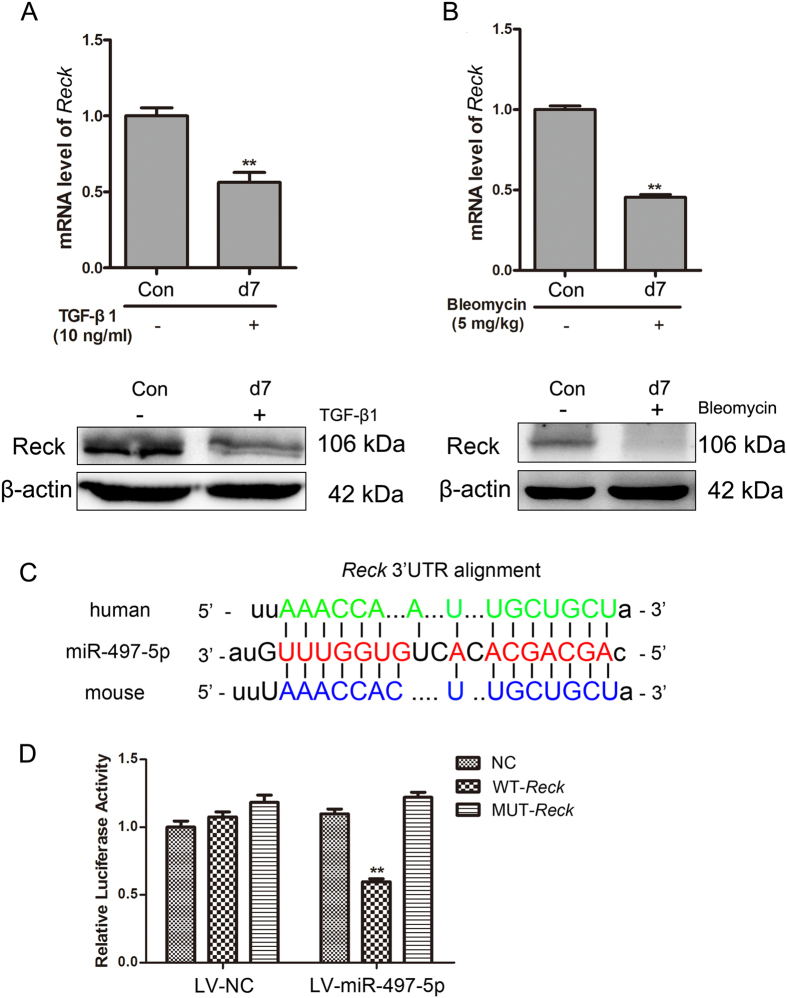
Reck is downregulated *in vitro* and *in vivo* as a target gene of miR-497-5p. (**A**) Lung resident mesenchymal stem cells (LR-MSCs) were incubated with TGF-β1 for 7 days. The level of Reck was determined by Western blot and quantitative real-time polymerase chain reaction. (**B**) Mice were administered with bleomycin, and the Reck expression of lung tissues was measured by Western blot and quantitative real-rime polymerase chain reaction. (**C**) The position of the miR-497-5p target site in the *Reck* 3′-UTR and the sequence alignment of miR-497-5p and the *Reck* 3′-UTR from various species are shown. (**D**) LV-miR-497-5p or negative control (LV-NC) was co-transfected with the mouse *Reck* 3′-UTR-derived GV306 vector, i.e. negative control (NC), wild type (WT-*Reck*) or mutated type (MUT-*Reck*) in the putative miR-497-5p seed region, into 293 T cells. The cells were harvested 48 h after transfection, and luciferase activity was analyzed. The values are presented as the means ± SD. The experiments were performed three times with similar results. **P < 0.01.

Reck is predicted to have a potential conserved binding site with miR-497-5p. We investigated the expression of Reck in TGF-β1-treated LR-MSCs and lung tissues administered with BLM. The results showed that Reck was dramatically suppressed (Fig. 2A and B), indicating a potential relationship between Reck and miR-497-5p.

### miR-497-5p regulates the differentiation of LR-MSCs by targeting *Reck*

The seed sequence of miR-497-5p within the 3′-UTR sequence of mouse *Reck* was predicted to be a potential conserved binding site (Fig. 2C). To confirm whether miR-497-5p could regulate *Reck* expression, *Reck* 3′-UTR was cloned into a luciferase reporter system (the GV306 vector). A *Reck* 3′-UTR mutant with mutations in the predicted miR-497-5p site was also cloned into a GV306 vector. The constructs were subsequently transfected into 293 T cells with either LV-miR-497-5p or LV-NC. Co-transfection with LV-miR-497-5p, which contained WT-*Reck* but not MUT-*Reck*, evidently diminished the normalized luciferase activity indicating that miR-497-5p could bind with the 3′-UTR of *Reck* and suppress the transcription of luciferase (Fig. 2D).

**Figure 3:**
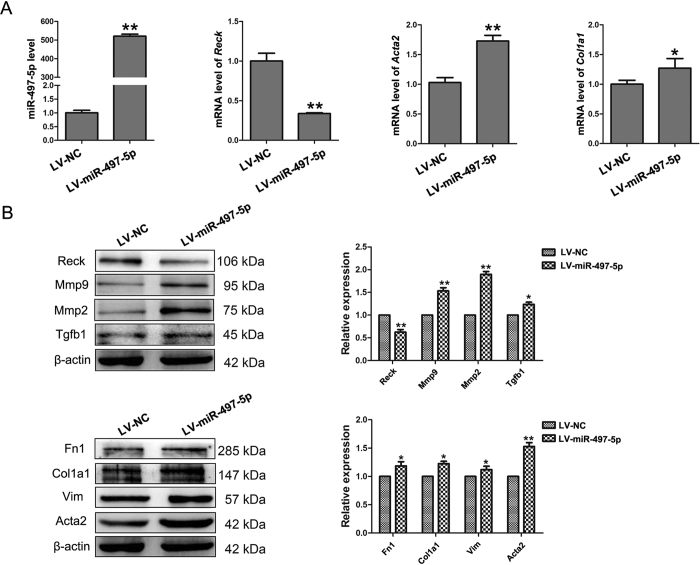
miR-497-5p induces the myofibroblast differentiation of lung resident mesenchymal stem cells (LR-MSCs). (**A**) LR-MSCs were transfected with either 5 × 10^7^ TU/ml negative control (LV-NC) or LV-miR-497-5p. At day 7, the levels of miR-497-5p, *Reck*, A*cta2* and Col1a1 were measured by quantitative real-rime polymerase chain reaction. Three independent experiments were performed, and the values are expressed as the means ± SD. *P < 0.05 and **P < 0.01. (**B**) The experiments were performed as in (**A**). The expression of Reck, Mmp2, Mmp9, Tgfb1, Fn1, Col1a1, Vim and Acta2 were determined by Western blot. The expression levels of protein were quantified by densitometry and normalized to the expression of β-actin. Three repeats were performed. *P < 0.05 and **P < 0.01.

**Figure 4:**
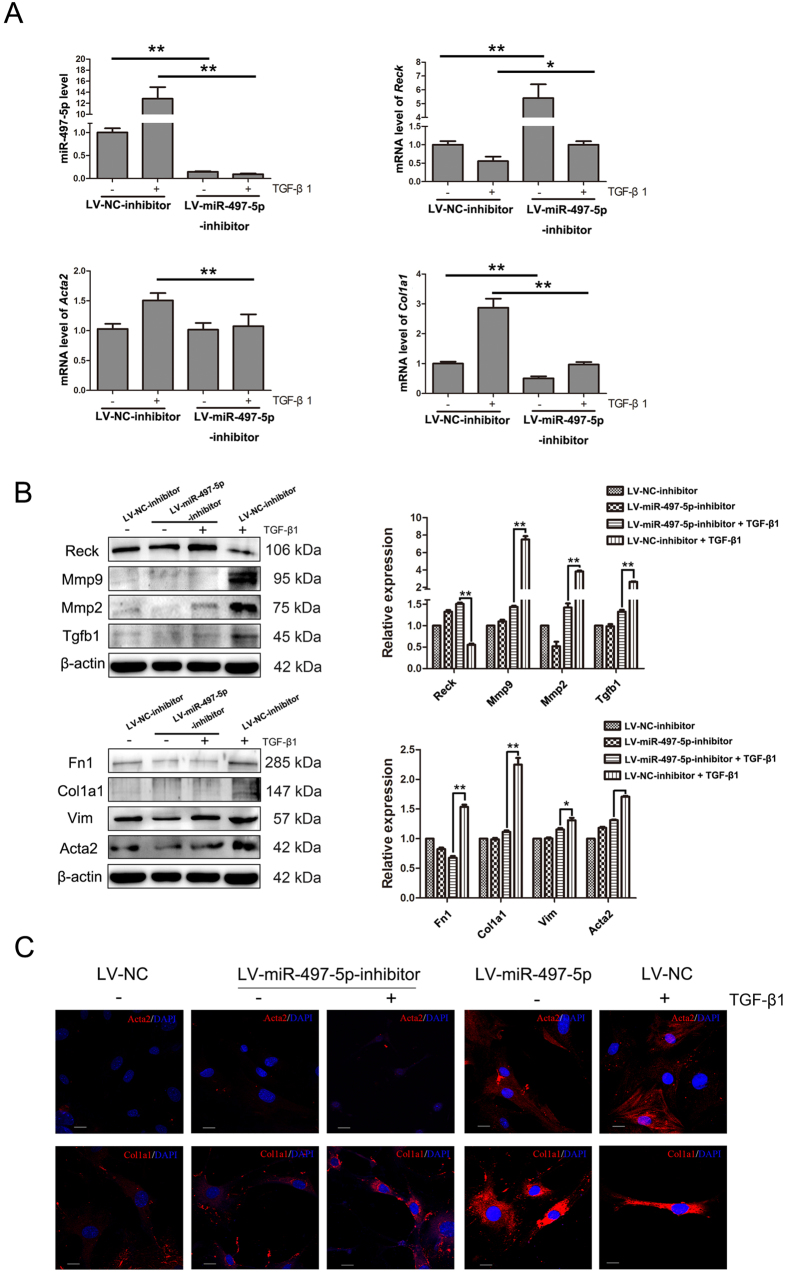
Inhibition of miR-497-5p suppresses TGF-β1-induced myofibroblast differentiation of lung resident mesenchymal stem cells (LR-MSCs). (**A**) LR-MSCs were transfected with either 5 × 10^7^ TU/ml negative control inhibitor (LV-NC-inhibitor) or miR-497-5p inhibitor (LV-miR-497-5p-inhibitor), followed with treatment either with or without 10 ng/ml TGF-β1 for 7 days. The levels of miR-497-5p, *Reck, Acta2* and *Col1a1* were measured by quantitative real-rime polymerase chain reaction. Three independent experiments were performed, and the values are expressed as the means ± SD. *P < 0.05 and **P < 0.01. (**B**) The experiments were performed as in (**A**). The expression of Reck, Mmp2, Mmp9, Tgfb1, Fn1, Col1a1, Vim and Acta2 was determined by Western blot. The expression levels of protein were quantified by densitometry and normalized to the expression of β-actin. Three repeats were performed. *P < 0.05 and **P < 0.01. (**C**) LR-MSCs were treated as explained in (Figs 3A and 4A), the expression of Acta2 and Col1a1 was measured by immunofluorescence. Acta2 and Col1a1were revealed with secondary 594-labeled antibodies; nuclei were revealed by DAPI staining.

To further explore if miR-497-5p upregulation was required for the myofibroblast differentiation of LR-MSCs, these cells were transfected with either LV-miR-497-5p or LV-NC followed by culture for 7 days. As shown in Fig. 3A and B, upregulating miR-497-5p could downregulate the expression of Reck, resulting in increased protein levels of Mmp2, Mmp9 and Tgfb1. In addition, overexpression of miR-497-5p could also induce α- smooth muscle actin (Acta2) and collagen I (Col1a1) expression at both mRNA and protein levels compared with LV-NC, along with increased protein levels of Vim (Fig. 3A and B). When LR-MSCs were transfected with either LV-miR-497-5p-inhibitor or LV-NC-inhibitor and cultured for 72 h followed by treatment with TGF-β1 for another 7 days, downregulation of miR-497-5p in LR-MSCs could decrease TGF-β1-induced Acta2, Vim and Col1a1 expression by upregulating the expression of Reck, resulting in decreased levels of Mmp2, Mmp9 and Tgfb1 (Fig. 4A and B). We also confirmed these findings using immunofluorescence staining (Fig. 4C). These data suggested that miR-497-5p was sufficient to modulate the differentiation of LR-MSCs and these cells play an important role in pulmonary fibrogenesis^26^.

### miR-497-5p induces myofibroblast differentiation of NIH/3T3 cells

**Figure 5:**
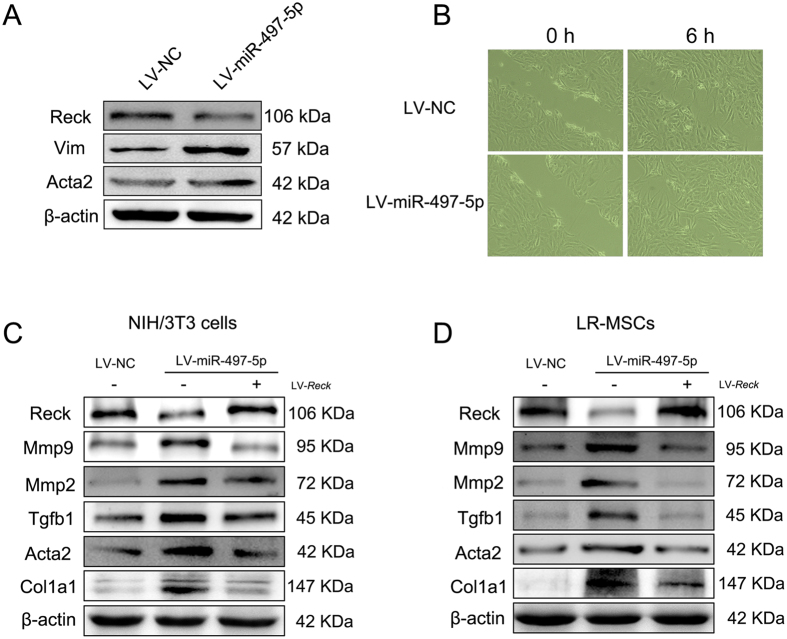
miR-497-5p induces myodifferentiation and migration of fibroblasts. (**A**) Mouse fibroblasts (NIH/3T3 cells) were transfected with either 5 × 10^7^ TU/ml of negative control (LV-NC) or LV-miR-497-5p. At day 7 after transfection, the levels of Vim, Reck and Acta2 were measured. (**B**) Fibroblast migration was measured after transfection. (**C**) NIH/3T3 cells were transfected with either 5 × 10^7^ TU/ml of LV-NC or LV-miR-497-5p. Some of the cells were co-transfected the pGCL mutant *Reck* (LV-*Reck*). The levels of Acta2, Col1a1, Reck, Mmp2, Mmp9 and Tgfb1 were measured by Western blot. (**D**) Lung resident mesenchymal stem cells were treated as in (**C**). The levels of Acta2, Col1a1, Reck, Mmp2, Mmp9 and Tgfb1 were measured by Western blot. Data represented three independent experiments.

As miR-497-5p was also increased in the lung tissues of the pulmonary fibrosis model, we also sought to illustrate whether miR-497-5p could induce myofibroblast differentiation of fibroblasts as this process is believed to play a key role in the development of IPF. In NIH/3T3 cells, miR-497-5p could also impair Reck expression and augment the levels of Acta2 and Vim, suggesting the myofibroblast differentiation of NIH/3T3 cells (Fig. 5A). The migration assay showed that miR-497-5p could promote the migration of NIH/3T3 cells (Fig. 5B). In order to further confirm the regulation of *Reck* by miR-497-5p, we transfected NIH/3T3 cells with miR-497-5p and a mutated form of *Reck* that had a disrupted 3′-UTR sequence followed by functional analysis. Our results found that such co-transfection suppressed miR-497-5p-induced downregulation of Reck and upregulation of Mmp2, Mmp9 and Tgfb1, counteracting miR-497-5p-induced myofibroblast differentiation of NIH/3T3 cells evidenced by reduced levels of Acta2 and Col1a1 (Fig. 5C). We also reproduced this finding in LR-MSCs (Fig. 5D). Taken together, these data strongly suggest that the profibrotic effect of miR-497-5p was meditated by binding with its target gene Reck.

### Intratracheal delivery of LV-miR-497-5p-inhibitor alleviates BLM-induced pulmonary fibrosis

**Figure 6:**
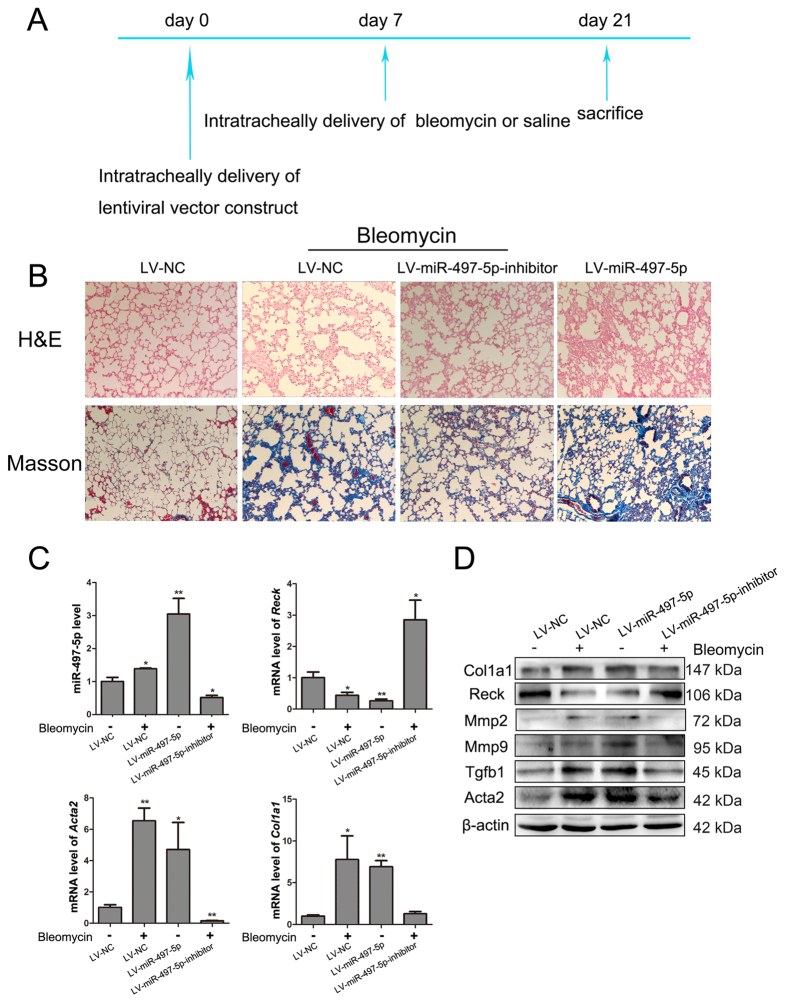
miR-497-5p regulates fibrogenesis in a mouse pulmonary fibrosis model. (**A**) A paradigm of the experiment for miR-497-5p lentivirus treatment is shown. Lentiviral vector was delivered intratracheally at day 0. After 7 days, mice were treated with bleomycin or saline intratracheally. Mice were sacrificed at day 21 after lentiviral vector instillation. (**B**) The lung sections were stained using H&E (100x magnification) and Masson’s trichrome (100x magnification). (**C**) Expression of miR-497-5p, *Reck, Acta2* and *Col1a1* was determined by quantitative real-rime polymerase chain reaction. (**D**) Levels of Col1a1, Reck, Mmp2, Mmp9, Tgfb1 and Acta2 were measured by Western blot. Data represented three independent experiments.

**Figure 7:**
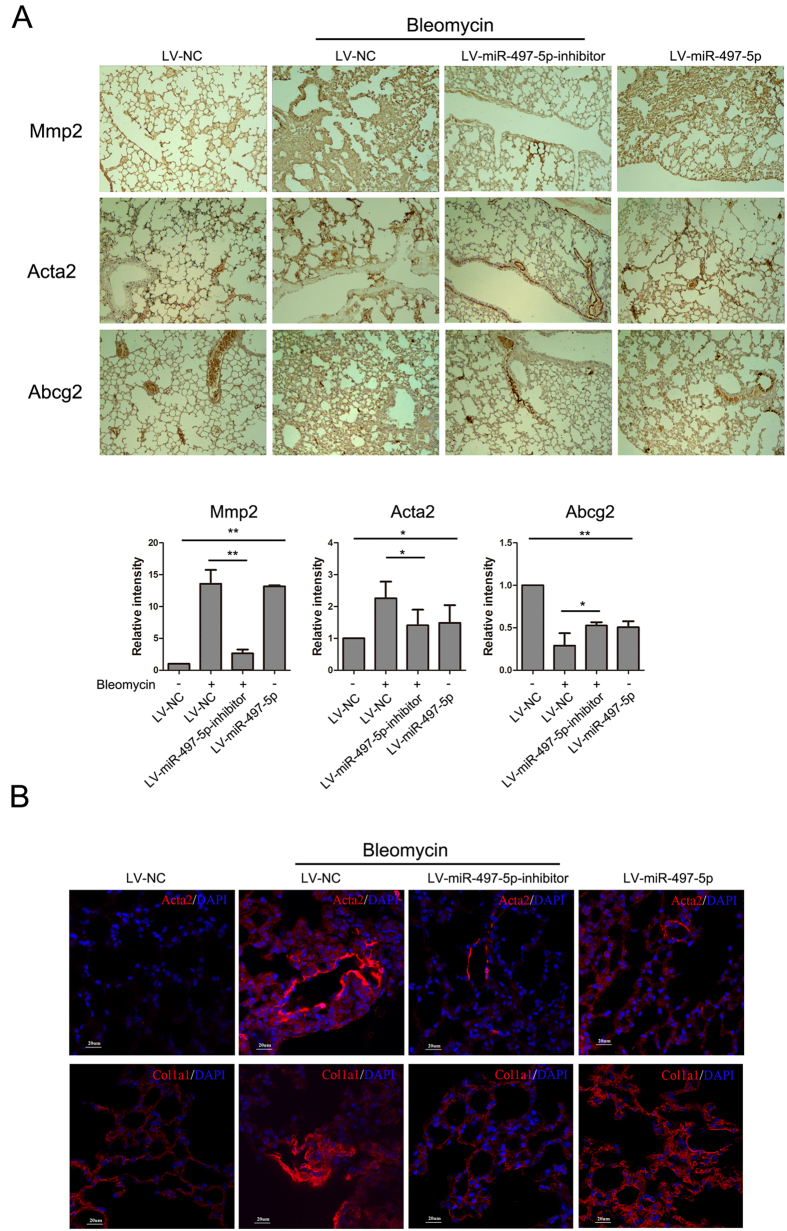
Inhibition of miR-497-5p suppresses bleomycin-induced pulmonary fibrosis. Mice were treated as in Fig. 6. (**A**) Immunohistochemistry assays were performed to measure Mmp2, Acta2 and Abcg2 expression (100x magnification). The positive areas were quantified by densitometry and normalized to LV-NC. (**B**) Expression of Acta2 and Col1a1 was determined by immunofluorescence staining. Acta2 and Col1a1 was revealed with secondary 594-labeled antibodies; nuclei were revealed by DAPI staining. The results shown are representative of three independently performed experiments.

Having demonstrated the profibrotic effect of miR-497-5p *in vitro*, we next explored whether regulating miR-497-5p expression may modulate the process of pulmonary fibrosis *in vivo*. To address this question, we administered intratracheally either a LV-NC or LV-miR-497-5p or LV-miR-497-5p-inhibitors to mice. At 7 days after treatment, the mice were treated intratracheally with BLM or saline. At day 21, the mice that were treated with LV-miR-497-5p-inhibitor exhibited decreased lung fibrosis as determined by histological analyses of the lungs and the Masson’s trichrome assay for collagen deposition (Fig. 6A and B). Furthermore, LV-miR-497-5p-inhibitor blocked BLM-induced miR-497-5p upregulation, while suppressed the mRNA expression of A*cta2* and *Col1a1* by elevating its target gene *Reck* (Fig. 6C). Downregulation of miR-497-5p also dampened Mmp2, Mmp9, Tgfb1, Acta2 and Col1a1 protein expression by impairing BLM-induced Reck downregulation (Figs 6D and 7A). LV-miR-497-5p-inhibitor could protect cells that express ATP-binding cassette transporter subtype G 2 (Abcg2), a marker of LR-MSCs, from degradation (Fig. 7A). On the contrary, when miR-497-5p was upregulated *in vivo*, miR-497-5p could promote pulmonary fibrogenesis (Figs 6 and 7). These results were also supported by immunofluorescence staining (Fig. 7B). Taken together, our work suggests that miR-497-5p can be used as a potential therapeutic target for pulmonary fibrosis.

## Discussion

**Figure 8:**
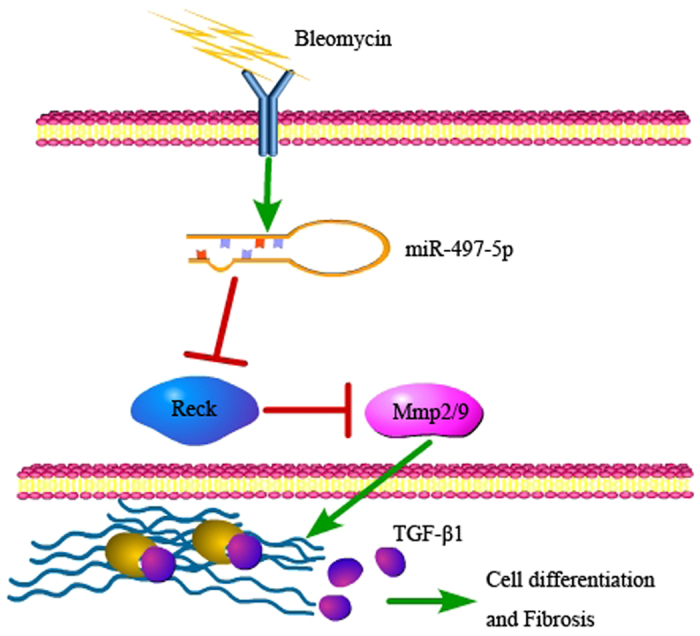
Proposed mechanism of pulmonary fibrosis induced by upregulated miR-497-5p. miR-497-5p targets *Reck*, which leads to myofibroblast differentiation of lung resident mesenchymal stem cells and pulmonary fibrosis through activating latent TGF-β1.

Crucial new insights into the diagnosis, treatment, prognosis and pathogenesis of various human diseases, including tissue fibrosis, have been provided by genome-wide approaches to miRNA expression profiling^17^,^27^,^28^. The application of these approaches in IPF has demonstrated that let-7d, miR-21 and miR-199a-5p make critical contributions to pulmonary fibrosis^15^,^21^,^29^. In our study, we explored the expression, regulation, and potential role of miRNAs in the myofibroblast differentiation of LR-MSCs. Among these significantly upregulated miRNAs, we focused on miR-497-5p and observed its high expression in the lung tissues of a pulmonary fibrosis model. miR-497-5p is one of the members of the miR-15/107 group with the seed sequence AGCAGC, which is an important determinant of target recognition^30^. It has previously been reported that miR-497-5p is closely related with several cancers^31^,^32^. However, the relationship between miR-497-5p and fibrosis remains unknown. In our investigation, we demonstrated that miR-497-5p could most likely bind with the 3′-UTR of *Reck* by the luciferase assay, strongly suggesting that *Reck* was a target gene of miR-497-5p. Additionally, we have demonstrated that upregulation of miR-497-5p induced myofibroblast differentiation of LR-MSCs and NIH/3T3 cells *in vitro*. We also found that the profibrotic function of miR-497-5p was mainly mediated through the activation of latent TGF-β1 anchored in ECM by targeting the 3′-UTR of *Reck*, which negatively regulates, at least three different Mmps, namely, membrane-type 1 matrix metalloproteinase (MT1-Mmp), Mmp2 and Mmp9 (Fig. 8). Secreted Mmps (e.g. MT1-Mmp, Mmp2 and Mmp9) have been implicated in the release and activation of TGF-β^13^,^33^,^34^, and may also be involved in the deregulation of the synthesis and degradation of ECM protein, a process that leads to enlarged ECM deposition in fibrosis^11^.

Pulmonary fibrosis is characterized by increased deposition of ECM and aberrant fibroblast proliferation^35^. LR-MSCs undergo injury-induced phenotypic modulation to become α-SMA positive myofibroblasts, which is a crucial step in the repair process that facilitates collagen secretion following lung injury. The collagen deposition and the proliferation of myofibroblasts at the site of injury result in scar formation, which helps to maintain alveolar structural integrity and function^36^. Activation of the TGF-β1 pathway is a significant event in the fibrogenic response, as it contributes to myofibroblast differentiation of LR-MSCs and pulmonary fibroblasts, and triggers the synthesis of ECM proteins^37^,^38^. *In vivo*, intratracheal inhalation of a miR-497-5p hairpin inhibitor was sufficient to induce the expression of Reck and decrease the release of activated TGF-β1 by suppressing Mmp2 and Mmp9 expression, which attenuated the fibrotic process. In contrast, upregulating the expression of miR-497-5p could induce the expression of Acta2 and Col1a1 and contribute to the phenotype of pulmonary fibrosis by activating the Mmps/TGF-β pathway.

Moreover, as miRNAs could regulate more than one mRNA, some other miR-497-5p-predicted target genes, such as Smad7, a negative regulator of TGF-β signaling^39^, were also implicated in the phenotype of pulmonary fibrosis^40^. Considerable data have suggested that miRNAs play key roles in the development of pulmonary fibrosis and may be explored as promising therapeutic targets for pulmonary fibrosis. For example, Liu *et al*. demonstrated that miR-21 is overexpressed in the lungs of both mice with BLM-induced fibrosis and patients with IPF^21^. Pandit *et al*. found that let-7d is downregulated in the lungs of IPF patients and the number of epithelial cells that express let-7d is correlated with pulmonary function^15^. In addition, miR-29, miR-31 and miR-200 each plays an anti-fibrotic role in the lungs^41^,^42^,^43^. However, prior to our study, no experimental evidence had shown that miR-497-5p could contribute to lung fibrosis. To our knowledge, our current work represents the first report arguing that administration of a miR-497-5p inhibitor may attenuate the fibrotic processes both *in vitro* and *in vivo*. Our data supported that miR-497-5p modifying drugs may have a therapeutic benefit for IPF.

## Materials and Methods

### Ethics statement

The animal experiments were performed according to the Guide for the Care and Use of Laboratory Animals (The Ministry of Science and Technology of China, 2006) and all experimental protocols were approved under the animal protocol number SYXK (Su) 2009-0017 by the Animal Care and Use Committee of Nanjing University.

### miRNA microarray

Total RNA was isolated from LR-MSCs incubated with TGF-β1 (PeproTech, Rocky Hill, NJ) for 7 days using Trizol Reagent (Ambion, Foster, CA). miRNA profiling was performed using a low-density miRNA Taqman array service (Invitrogen, Shanghai, China). The miRNAs exhibiting an expression fold change (log_2_) greater than 1 or less than -1 were deemed to be differentially expressed. We validated a series of abundantly and differentially expressed miRNAs via quantitative reverse transcription polymerase chain reaction (Q-RTPCR).

### Antibodies

Mouse monoclonal antibody against mouse β-actin (ab8277), mouse monoclonal antibody against mouse Mmp2 (ab86607), rabbit polyclonal antibody against mouse Mmp9 (ab38898), rabbit polyclonal antibody against mouse Acta2 (ab5694), rabbit monoclonal antibody against mouse Vim (ab92547), rabbit polyclonal antibody against mouse Col1a1 (ab34710), mouse monoclonal antibody against mouse Tgfb1 (ab27969), and rabbit polyclonal antibody against mouse Fn1 (ab2413) were purchased from Abcam (Cambridge, MA). Rabbit monoclonal antibody against mouse Reck (D8C7) was purchase from Cell Signaling Technology (Beverly, MA).

### Cell culture

Mouse fibroblast cells (NIH/3T3) was obtained from the American Type Culture Collection (Manassas, VA). The cells that were frozen down at an early passage were cultured for a maximum of eight passages. The cells were maintained at 37 °C with 5% v/v CO_2_ in Dulbecco’s modified Eagle’s medium (DMEM, Life Technologies/Gibco, Grand Island, NY) supplemented with 10% fetal bovine serum (FBS, Gibco).

Isolation of LR-MSCs was performed as previously reported^44^. Freshly isolated LR-MSCs were cultured at a concentration higher than 10^5^ cells/ml with DMEM (Gibco) containing 15% FBS (Gibco), 4% L-glutamine, 1% nonessential amino acids, and 1% penicillin and streptomycin, and maintained in a humidified atmosphere of 95% air, 5% CO_2_ at 37 °C. The cells were passaged 1:2 using 0.25% trypsin when they reached 70-90% confluence.

### Vector transfection

Cells at 60% confluence were transfected with lentiviral vectors at the concentration of 5 × 10^7^ TU/ml that contained the GV369 miRNA precursor of miR-497-5p (LV-miR-497-5p), the GV280 miR-497-5p inhibitor (LV-miR-497-5p-inhibitor) or the pGCL mutant *Reck* (LV-*Reck*). In all experiments, an equal concentration of non-targeting sequence GV369 miRNA precursor negative control (LV-NC), GV280 miRNA inhibitor negative control (LV-NC-inhibitor) or the pGCL mutant *Reck* negative control was used. The lentiviral vector was purchased from GENECHEM (Shanghai, China).

### Luciferase assays

For luciferase assays, the sequence of the *Reck* 3′-UTR and the *Reck* 3′-UTRs in which the putative binding sites had been mutated were amplified with specific primers, and verified by DNA sequencing. These gene fragments were then subcloned into the GV306 vector (GENECHEM) to generate the wild-type *Reck* plasmid (WT-*Reck*) and the mutant *Reck* plasmid (MUT-*Reck*). 293 T cells were transiently co-transfected with 125 ng GV306 vector and 5 × 10^7^ TU/ml LV-miR-497-5p or LV-NC lentivival vector. Luciferase assays were performed 48 h later using the Dual-Luciferase Reporter System (Promega, Madison, WI). The Renilla and firefly luciferase signals were detected using a GloMax^®^-Multi + Detection System (Promega). The activity of an internal firefly luciferase was normalized by the Renilla luciferase activity.

### Induction and treatment of pulmonary fibrosis

All animal procedures were conducted in accordance with humane animal care standards approved by the Nanjing University Ethics Committee (Nanjing, China) and maintained under specific pathogen-free conditions. The animals were acclimated to the environment for 1 week prior to treatment.

The mice were administered with BLM (Nippon Kayaku, Tokyo, Japan) intratracheally at a dose of 5 mg/kg dissolved in a total of 50 μl sterile saline. The control group was similarly treated with 50 μl of sterile saline. The lentiviral vector of LV-miR-497-5p and LV-NC (LV-miR-497-5p-inhibitor and LV-NC-inhibior, respectively) were purchased from GENECHEM. Mice were administered with the lentiviral vector intratracheally at a dose of 2 × 10^8^ TU/ml diluted by sterile saline. Seven days later, the LV-miR-497-5p-inhibitor and LV-NC-inhibitor groups were administered with BLM intratracheally at a dose of 5 mg/kg dissolved in a total of 50 μl sterile saline. The LV-miR-497-5p and LV-NC groups were similarly treated with 50 μl sterile saline. The mice were sacrificed for lung collection at day 14 after BLM administration (n = 6 for each time point).

### SDS-PAGE and immunoblotting

Briefly, whole cell or tissue lysates were separated on 12% SDS-polyacrylamide gels and transferred to a polyvinylidene fluoride (PVDF) membrane (Roche, Germany) by standard procedures. Membranes were blocked by incubation for 1 h with 5% non-fat milk in PBS containing 0.5% Tween-20 (PBST) and blotted with specific antibodies at 4 °C for 12 h. After three washes in PBST, the membranes were incubated with the secondary antibody at 37 °C for 1 h. Immunoreactive protein bands were detected using an Odyssey Scanning System (LI-COR, Lincoln, NE).

### Q-RTPCR

For Q-RTPCR analysis of mRNAs and mature miRNAs, total RNA was extracted using Trizol Reagent (Ambion). An amount of 0.05 μg total RNA was reverse-transcribed using the Taqman MicroRNA Reverse Transcription Kit (Applied Biosystems, Foster, CA). Comparative quantitative PCR (Q-PCR) was performed in triplicate using Taqman Universal PCR Master Mix (Applied Biosystems) on the 7300 Real-Time PCR System (Applied Biosystems). Mature miR-497-5p probes were alternatively obtained from Applied Biosystems. Normalization was performed by using RNU6B probes (Applied Biosystems). Relative expression was calculated by using the comparative Ct (ΔΔCt) method.

For analysis of mRNA, HiScript 1st Strand cDNA Synthesis Kit (Vazyme, Nanjing, China) was used for reverse transcription polymerase chain reaction (RT-PCR). Q-PCR was performed using the SYBR Green Q-PCR Kit (Roche, Germany). Specific primers for mRNAs are listed in Table 1. The Ct values were analyzed using the ΔΔCt method and relative changes of mRNA levels were obtained by normalization to glyceraldehyde-3-phosphate dehydrogenase (GAPDH) relative to the control.

### Histopathology

The mouse lungs were inflated with a neutral buffered formalin solution overnight and embedded in paraffin before sectioning into 5 μm-thick slices. The sections were stained with hematoxylin-eosin (H&E) and Masson’s trichrome to assess the degree of fibrosis.

### Immunohistochemistry

Five μm-thick paraffin-embedded sections were deparaffinized with xylene (twice for 5 minutes each) before being rehydrated in water using an ethanol gradient. After washing with water, antigen retrieval was performed in a steamer using citrate buffer (pH 6.0, DAKO) for 20 minutes, and the samples were then cooled to room temperature. The sections were then washed with PBST, incubated with 3% H_2_O_2_ for 10 minutes and blocked with the avidin/biotin blocker and the serum-free blocking reagent. The sections were subsequently incubated with mouse anti-Mmp2, rabbit anti-Mmp9, rabbit anti-Abcg2 or rabbit anti-Acta2 antibodies overnight at 4 °C. The DAB Substrate System (DAKO) was used to reveal the immunohistochemical staining.

### Immunofluorescent staining

The immunofluorescence analysis was performed as described^44^. Rabbit anti-Col1a1 and rabbit anti-Acta2 were employed as the primary antibody. Alexa Fluor 594-conjugated goat anti-rabbit IgG (Invitrogen) was used as the secondary antibody. Nuclei were stained with 1 μg/ml DAPI (Sigma). The images were captured using a confocal fluorescence microscope (Olympus, Tokyo, Japan).

### Statistical Analysis

The data are presented as mean values ± SD. Differences were analyzed for significance (P < 0.05) by one-way ANOVA using SPASS for windows version 11.0 (SPASS, Chicago, IL).

### Supplement files for low-density miRNA Taqman array

The raw data of profiled miRNA in lung resident mesenchymal stem cells following TGF-β1-induced myofibroblast differentiation was uploaded in the supplement file.

## Additional Information

**How to cite this article**: Chen, X. *et al*. The role of miR-497-5p in myofibroblast differentiation of LR-MSCs and pulmonary fibrogenesis. *Sci. Rep.* **7**, 40958; doi: 10.1038/srep40958 (2017).

**Publisher's note:** Springer Nature remains neutral with regard to jurisdictional claims in published maps and institutional affiliations.

## Figures and Tables

**Table 1:** Q-RTPCR primers and products.

Genes	Forward primer	Reverse primer
*Reck*	AGATAACCAAATGTGCCGTGAT	TCAACCATTGTTTCAGGGCAATA
*Acta2*	CCCAGATTATGTTTGAGACCTTC	ATCTCCAGAGTCCAGCACAATAC
*Col1a1*	CTTCTGGTCCTCGTGGTCTCCCT	AAGCCTCGGTGTCCCTTCATTCC
*Gapdh*	AGGTCGGTGTGAACGGATTTG	TGTAGACCATGTAGTTGAGGTCA
